# Insecticide-treated net utilization and associated factors among pregnant women and under-five children in East Belessa District, Northwest Ethiopia: using the Health Belief model

**DOI:** 10.1186/s12936-021-03666-6

**Published:** 2021-03-04

**Authors:** Amlaku Nigusie Yirsaw, Resom Berhe Gebremariam, Wallelign Alemnew Getnet, Muhabaw Shumye Mihret

**Affiliations:** 1 Department of Health Education and Behavioral Sciences, Institute of Public Health, College of Medicine and Health Sciences, University of Gondar, P. O. Box 196, Gondar, Ethiopia; 2 Department of Clinical Midwifery, School of Midwifery, College of Medicine and Health Sciences, University of Gondar, Gondar, Ethiopia

**Keywords:** Ethiopia, Health belief model, Insecticide-treated net, Malaria, Utilization

## Abstract

**Background:**

Malaria during pregnancy and childhood is one of the major public health challenges globally. Its prevalence is huge in Africa, especially in sub-Saharan countries and Ethiopia. Insecticide-treated mosquito net (ITN) use is one of the primary malaria preventive strategies. Previous studies did not adequately address the health belief and behaviour-related correlates of ITN using health belief model (HBM), although a number of studies were conducted in this theme. Therefore, this study was aimed at assessing the prevalence and associated factors of ITN utilization among pregnant women and under five children in east Belessa district, northwest Ethiopia, 2020.

**Methods:**

A community-based cross-sectional mixed study was conducted in east Belessa district from February 01–30/2020. A total of 724 eligible participants were included in the quantitative study. A multistage cluster sampling technique was used. The quantitative data were collected using an interviewer-administered structured questionnaire. Data were entered into Epi data version 4.6.0.2 and then exported to SPSS version 16 for analysis. The binary logistic regression model was fitted and the level of significance was declared based on AOR with its 95% CI and p-value ≤ 0.05. Meanwhile, the qualitative data were collected using focus group discussions and key informant interviews, and analysed using a thematic analysis approach.

**Results:**

The prevalence of ITN utilization was 56.5% (95% CI 53.0, 60.2) and independently predicted by a corrugated iron roof of the house (AOR = 1.53; 95% CI 1.15, 2.22), rural residence (AOR = 1.59; 95% CI 1.11,2.28), ≥ 2 number of rooms in the house (AOR = 1.56; 95% CI 1.06, 2.30) and high level of perceived barrier (AOR = 0.53; 95% CI 0.38,0.74). In the qualitative findings, the main barrier was connected to misconceptions and misperception towards malaria and ITN.

**Conclusion:**

The prevalence of ITN utilization in the study area was lower than the national target (100%). It was significantly associated with household characteristics, residence, and level of a perceived barrier. Reversing the community’s misconceptions through information, education and communication (IEC), and behavioural change communication (BCC) would enhance ITN utilization.

## Background

Malaria is a common and life-threatening disease across the globe with higher burden in tropical and sub-tropical countries, including Ethiopia [1]. The incidence of malaria infection has been declined globally since 2010. However, its incidence and fatality rate remains steady in Africa. In 2018, the estimated 228 million malaria cases and 405,000 deaths from malaria were reported globally. Of these, Africa accounts 93% of the cases and 94% of the malaria deaths. Malaria is also among the major indirect causes of maternal and under-five children mortality [2–4].

Insecticide-treated bed net (ITN) utilization is one of the effective malaria preventive strategies. It can prevent malaria infection by reducing the contact and bite of mosquitoes if it is employed properly and consistently. Evidence shows that ITN utilization can reduce malaria related deaths by about 20% from all causes of mortality in malaria-endemic areas [5]. Studies examining ITN effectiveness point out a reduction in malaria episodes by 48–50% [6]. If used universally, about 7% of global under-five mortality could be prevented. In addition to the direct benefit to the individual, ITN utilization gives the community an indirect protective benefit. Expanding ITN utilization in a community is far effective than focusing on ITN utilization by individuals [7].

Many articles indicate that ITN utilization among pregnant women (PW) and under five children (UFC) in sub-Saharan countries is very low [8–10]. Poor educational and awareness level, and ITN related factors (i.e., ITN accessibility, sufficiency, quality, physical condition, maintenance, replacement and effectiveness) contribute to poor ITN utilization [11–15]. However, these factors could vary across settings and over time. Moreover, previous studies did not adequately investigate on the health belief and behaviour-related factors using the Health Belief Model (HBM).

The HBM is one of the most widely used conceptual frameworks in health behaviour research to explain changes and maintenance of health-related behaviours, as well as to guide health behavioural interventions. The HBM assumes that if the person believes that a negative health condition can be avoided, he/she will take a health-related action. Conversely, the model assumes that if a person has a positive hope that he/she can prevent a negative health problem by taking a prescribed step, he/she will act accordingly [16]. HBM has six constructs namely perceived susceptibility, perceived severity, perceived benefit, perceived barrier, self-efficacy and cues to action [17]. Certain previous studies utilized HBM to explore participants’ perception towards malaria and ITN utilization. However, these studies reported inconsistent findings [17, 18]. For example, a study conducted in Myanmar revealed that almost all respondents had high perceived severity of malaria which implies that respondents could tend to use ITN [17]. In contrast, a similar study undertaken in Jimma, Ethiopia showed that majority of the participants had low perceived severity of malaria; this implies that respondents would not intend to use ITN. Furthermore, this study did not touch the qualitative aspects in spite of considering HBM [18]. Hence, limited evidence on the use of both HBM and qualitative study in connection to ITNs utilization, and inconsistent findings across literature is one of the questions yet to be answered. Moreover, to the best investigators’ knowledge, there was no study which had been conducted through both mixed study design and HBM to address the behavioural and perception-related factors on ITN utilization among PW and/or UFC in the rural communities of Ethiopia.

Therefore, the current study was aimed at assessing the prevalence and associated factors of ITN utilization, and the behavioural, perceptional and other related barriers of ITN utilization among PM and/or UFC in one of the malaria endemic districts in Amhara national regional state—East Belessa, northwest Ethiopia. In addition, doing the research may also provide basic information for programme managers and policy makers targeting on prevention of malaria infection and reduction of corresponding indirect maternal and children mortalities and/or morbidities. The findings may also form a significant reference material to researchers working on this theme.

## Methods

### Study design, period, settings and population:

A community-based cross-sectional study with a mixed approach of quantitative and qualitative methods was applied from February 01 to February 30, 2020. The study was conducted in east Belessa district, Amhara national regional state, northwest Ethiopia. East Belessa is one of the 15 districts in the central Gondar zone which is located 736 kms away from Addis Ababa—the capital city of Ethiopia, and 170 kms from Bahirdar—the capital city of Amhara national regional state. There are about 30 *kebeles* (i.e., the smallest administrative units) in east Belessa district. The estimated total population of the district was 144,815 as of a 2019 health statistics survey report of the district. Of whom, about 71,683 (49.5%), 4880 (3.7%) and 19,608 (13.54%) were females, PW and UFC, respectively. In the district, there are about 33,679 households. The district is serviced by 36 governmental health facilities including one primary hospital, five health centers, and 30 health posts; and three private clinics [19]. Geographically, 90% of the villages of the district are located in the lowland and the remaining 10% of the villages in the temperate zone. Hundred percent of the population in the district is at risk of malaria attack. In the study area, malaria was reported to be the leading cause of morbidities in 2019. By June 2019, 78,224 ITNs were distributed for nearly all households [20].

In this study, PW and/or UFC who have resided in the selected kebeles of the district were included. On the other hand, PW who have resided for < 6 months were excluded as people who have lived for < 6 months in a given administrative unit are not officially recognized as residents. In addition, UFC having non-resident care takers (i.e., who have dwelled for < 6 months) were excluded for the same reason.

### Sample size determination and sampling technique

The sample size was calculated to be 724 using Open-Epi Version 7 software by considering the following assumptions; prevalence of ITN utilization among PW and UFC—69% [21], level of confidence—95%, margin of error—5%, non-response rate, and design effect—2. A multi-stage cluster sampling technique was used to select the participants. First, we obtained a list of a total of 30 kebeles (clusters) in the district. Then, 7 (23%) of the total clusters were picked randomly. The data collectors then went from home to home and checked for the presence of eligible study participants in all households in each cluster. Thereafter, all eligible participants were provided information regarding the basic elements of informed consent and then requested to offer their consent for participation. After having a written informed consent, the data collectors performed two consecutive tasks—face to face interviews and direct observation for ITN utilization. The same procedures were undertaken by all data collectors for all eligible participants in the households within the selected clusters of a district. Upon the presence of two or more eligible participants in a household, one of them was selected randomly.

For the qualitative study, the participants were selected by the primary investigator purposively in consultation with the kebele leader among PW, UFC caregivers, and significant others. Accordingly, a total of 39 participants were assigned in five focus group discussion (FGD) groups. These participants were selected among members of urban women’s health development army, rural women’s health development army, male care givers of UFC, urban female care givers of UFC, urban PW, rural female caregivers of UFC and rural PW. In addition, Key Informant Interview (KII) was undertaken from four health extension workers and one woreda malaria officer.

### Variables of the study

The outcome variable for this study is ITN utilization which was dichotomized as “Yes”—coded to be “1” and “No”—coded to be 0, whereas, the explanatory variables include socio-demographic, environmental and awareness related variables such as age, gender, education status, marital status, occupation, residence, ethnicity, religion, housing condition and knowledge status. In addition, HBM constructs including perceived susceptibility, perceived severity, perceived benefit, perceived barrier, self-efficacy, and cues to action were included in the explanatory variables.

### Operational definitions

ITN utilization was measured based on respondents’ self-report together with direct observation. Accordingly, ITN utilization was recorded to be “yes” if a PW or a care giver of UFC reported that a PW and/or a child slept under ITN during the night prior to the survey date and ITN was observed to be hanged (mounted) over the bed/the sleeping area during the observation day. On the other hand, ITN utilization was labeled to be “no” if a PW or a care giver of UFC reported that a PW and/or a child did not sleep under ITN during the night prior to the survey date or if the ITN was not observed to be hanged (mounted) over the bed/the sleeping area during the observation day despite a positive participant’s report [22–25].

Perceived susceptibility refers to the perception of PW and care givers of UFC about the chance of getting a malaria infection and was assessed through 6 items of questions [16, 17]. It was classified as high perceived susceptibility (i.e., a score of ≥ the mean score) and low perceived susceptibility (i.e., a score of < the mean value) [17].

Perceived severity refers to the beliefs of the respondents about the seriousness or severity of the malarial disease and was assessed through 6 items of questions [16, 17]. It was classified as high perceived severity (i.e., a score of ≥ the mean value) and low perceived severity (i.e., a score of < the mean value) [17].

Perceived benefit refers to the participants’ feeling of quality or usefulness of using ITN in diminishing the danger of developing the malarial disease and was assessed through 6 items of questions [16, 17]. It was categorized as high perceived benefit (i.e., a score of ≥ the mean value) and low perceived benefit (i.e., a score of < the mean value) [17].

Perceived barrier refers to the perception of respondents about the difficulty of ITN utilization and was assessed through 6 items of questions [16, 17]. It was reported as high perceived barrier (i.e., a score of ≥ the mean value) and low perceived barrier (i.e., a score of < the mean value) [17].

Self-efficacy refers to the perception or confidence of respondents towards ITN utilization and was assessed through 6 items of questions [16, 17]. It was reported as high perceived self-efficacy (i.e., a score of ≥ the mean value) and low perceived self-efficacy (i.e., a score of < the mean value [17].

Cues to action refers to the participant’s cue or redness to initiate ITN utilization and was assessed through 6 items of questions [16, 17]. It was categorized as high perceived cues to action (i.e., a score of ≥ the mean value plus standard deviation) and low perceived cues to action (i.e., a score of < the mean score) [17].

Knowledge about malaria and IT was measured by using 25 items of knowledge assessing questions and categorized as good knowledge (i.e., a score greater ≥ the mean score) and poor knowledge (i.e., a score of < the mean score) [17].

### Data collection tools, methods and procedures

For the quantitative wing of the study, data were collected using pretested and structured questionnaire, which was adapted from related literature [17, 26, 27] through face to face interview. In addition, an observational checklist was used to check if the net has been hung over the bed or sleeping place. Seven unemployed graduates were recruited for the data collection process. These included six diploma clinical nurses for data collection and one public health professional for supervision. A one-day training was provided before the actual data collection commencement. Pre-test was done on 5% of the sample size. In addition, necessary revision was made on the tools after pretest. Data collectors were supervised every day and samples of respondents were re-interviewed and the results were then cross-checked. The questionnaires were first prepared in English, translated into Amharic (local) language and finally back to English. Each data collector checked the questionnaires for completeness before leaving each study participant. Each questionnaire was reviewed daily for completeness and clarity.

For the qualitative part, FGDs and KII have been undertaken after obtaining a written informed consent from each participant. A semi-structured discussion guideline, which encompassed three main themes including knowledge, perception and barriers towards ITN utilization, was utilized. Both FDG and KII were facilitated by the principal investigator. Audio and note recording were utilized. The recorded data were transcribed in Amharic and then translated into English.

### Data processing and analysis

#### Quantitative part

Data were checked, coded and entered into EpiData version 4.6.0.2. Data were also then exported to SPSS version 16 for analysis. Both descriptive and analytical statistical procedures were done and results were presented using tables and texts. Binary logistic regression model was used to identify factors associated with ITN utilization. Both bivariate and multivariable logistic regression analyses were carried out. All the variables in the crude analyses were included in the adjusted analyses as the total numbers of explanatory variables are only 14 (i.e., the number of variables are within the scope which the model can process) and all variables fulfilled the assumptions for Binary logistic regress model. Accordingly, the analysis was controlled for the variables including age, residence, educational status, occupation of the PW and caregivers of UFC, number of sleeping rooms in the house, number of peoples lived in the house, the roof of the house, knowledge, perceived susceptibly, perceived severity, perceived benefit, perceived barrier, self-efficacy and cues to action of the PW and caregivers of UFC. Both COR and AOR with the corresponding 95% CI were computed. Finally, the level of significance was declared based on AOR with its 95% CI and p-value ≤ 0.05. Model fitness was checked using Hosmer and Lemeshow goodness of fit test.

#### Qualitative data

An open code was employed for qualitative data analysis. First, recorded voices were transcribed and then read and re-read to develop codes that identify important and common concepts related to the main themes of the study. Second, the data were coded and categorized with respect to the main three themes including knowledge, perception and barriers of ITN utilization. Then, the data were analysed by sorting information, and looking for similarities, differences, or contradictions. Finally, the qualitative data were summarized and presented along the main three aforementioned themes to complement the quantitative data.

## Results

### Socio-demographic and housing characteristics of the respondents

A total of 724 respondents [i.e., 144 (19.9%) PW and 580 (80.1%) caregivers of UFC] participated in the quantitative study, yielding a response rate of 100%. About 66 (9.1%) of the respondents were males and 554 (76.5%) of the participants were rural dwellers. More than half (51.1%) of the participants were in the age group of 18–28-year-old with the mean ± standard deviation (SD) of 28.7 ± 5.7.

A majority (87.3%) of the respondents were married. About 687 (94.9%) of the participants were Orthodox Tewahido religious followers and all (100%) of the respondents were Amhara by ethnicity. About 400 (55.2%) of the participants had never attended any formal education and 531 (73.3%) of the respondents were housewives by occupation. Regarding the household’s characteristics, about 58.6% of the households sheltered more than four family members. Substantial (79.3%) of the households’ roof was made up of corrugated iron sheets. One hundred and sixty-nine (23.3%) of the households had at least two sleeping rooms with the mean number (± SD) of rooms per household of 1.23 (± 0.506) (Table 1).

**Table 1:** Socio demographic and household characteristics of the respondents in east Belessa, northwest Ethiopia, 2020

Variables	Number	%
Age of respondents		
18–28	370	51.1
29–39	321	44.3
40–50	30	4.2
51–61	3	0.4
Total	724	100
Marital status		
Married	632	87.3
Single	44	6.1
Divorced	42	5.8
Widowed	6	0.8
Total	724	100
Religious		
Orthodox Christian	687	94.9
Muslim	37	5.1
Total	724	100
Mother’s educational status		
No formal education	400	55.2
Grade 1–4	103	14.2
Grade 5–8	83	11.5
Grade 9–10	93	12.9
Grade 11–12	22	3
Certificate and above	23	3.2
Total	724	100
Mother’s occupation		
Housewife	531	73.3
Farmer	60	8.3
Daily labour	37	5.1
Government/NGO employee	22	3.1
Trader	74	10.2
Total	724	100
Number of family members		
< 4	300	41.4
≥ 4	424	58.6
Total	724	100
Roof of house		
Thatched-roof	150	20.7
Corrugated iron	574	79.3
Total	724	100
Total number of sleeping room		
One	555	76.7
Two and above	169	23.3
Total	724	100

### Knowledge about malaria and ITN

Two hundred and two (27.9%) of the respondents had low levels of knowledge. The overall mean value was 18 with minimum and maximum score of 6 and 22, respectively. A majority (82%) of the respondents knew that mosquito bites are causing malaria. Concerning the symptom of malaria, 98.3% of respondents reported chills (shivering) as a symptom of malaria followed by fever (96.4%), headache (86.5%), and sweating (83.8%). With respect to respondents’ knowledge on the malaria vulnerable groups: 91%, 82%, and 64% of the respondents reported the extremely malaria vulnerable groups of people to be UFC, PW and farmers, respectively. Moreover, most (97.5%) of the participants knew that ITN utilization could prevent malaria.

### Perception about malaria and ITN

#### Perceived susceptibility

Three hundred and seventy-three (51.5%) of the participants had low perceived susceptibility with the mean ± (SD) score of perceived susceptibility of 25.29 (± 3.79) (Table 2).

**Table 2:** Perception about malaria and ITN among respondents in east Belessa, Northwest Ethiopia, 2020

Theoretical variables	Poor	High	Total	Mean ± SD
Perceived susceptibility	373 (51.5%)	351 (48.5%)	724 (100%)	25.29 ± 3.79
Perceived severity	348 (48.1%)	376 (51.9%)	724 (100%)	25.78 ± 3.7459
Perceived benefit	341 (47.1%)	383 (52.9%)	724 (100%)	26.69 ± 3.4215
Perceived barrier	398 (55%)	326 (45%)	724 (100%)	11.5 ± 5.2942
Self-efficacy	398 (55%)	326 (45%)	724 (100%)	24.99 ± 3.7781
Cues to action	320 (44.2%)	404 (55.8%)	724 (100%)	22.57 ± 4.7024

#### Perceived severity

Three hundred and seventy-six (51.9%) of the respondents had high perceived severity of malaria with the mean (± SD) perceived severity score of 25.78 (± 3.7459) (Table 2).

#### Perceived benefit

About 96.1% of the respondents agreed that ITN utilization could prevent malaria. Three hundred and forty-one (47.1%) of the participants had low perceived benefits of ITN with the mean (± SD) perceived benefit score of 26.69 (± 3.4215) (Table 2).

#### Perceived barrier

Three hundred and ninety-eight (55%) of the respondents had low level of perception of the barrier of ITN with the mean (± SD) perceived barrier score of 11.5 (± 5.2942) (Table 2).

#### Self efficacy

Three hundred and twenty-six (45%) of the participants had high confidence of ITN utilization with the mean (± SD) self-efficacy score of 24.99 (± 3.7781) (Table 2).

#### Insecticide-treated bed net utilization

The prevalence of ITN utilization was 56.5% (95% CI 53.0, 60.2). About 78/144 (54%) of the PW and 360/580 (62.07%) of the UFC utilized ITNs. About 350 (48.3%), 265 (36.6%) and 109 (15.1%) of the households had only one, only two and ≥ three ITN, respectively. Seven hundred and twenty-four (100%) of the households had received at least one free ITN from the government within the past 12 months of the survey.

#### Barriers of ITN utilization

**Fig. 1:**
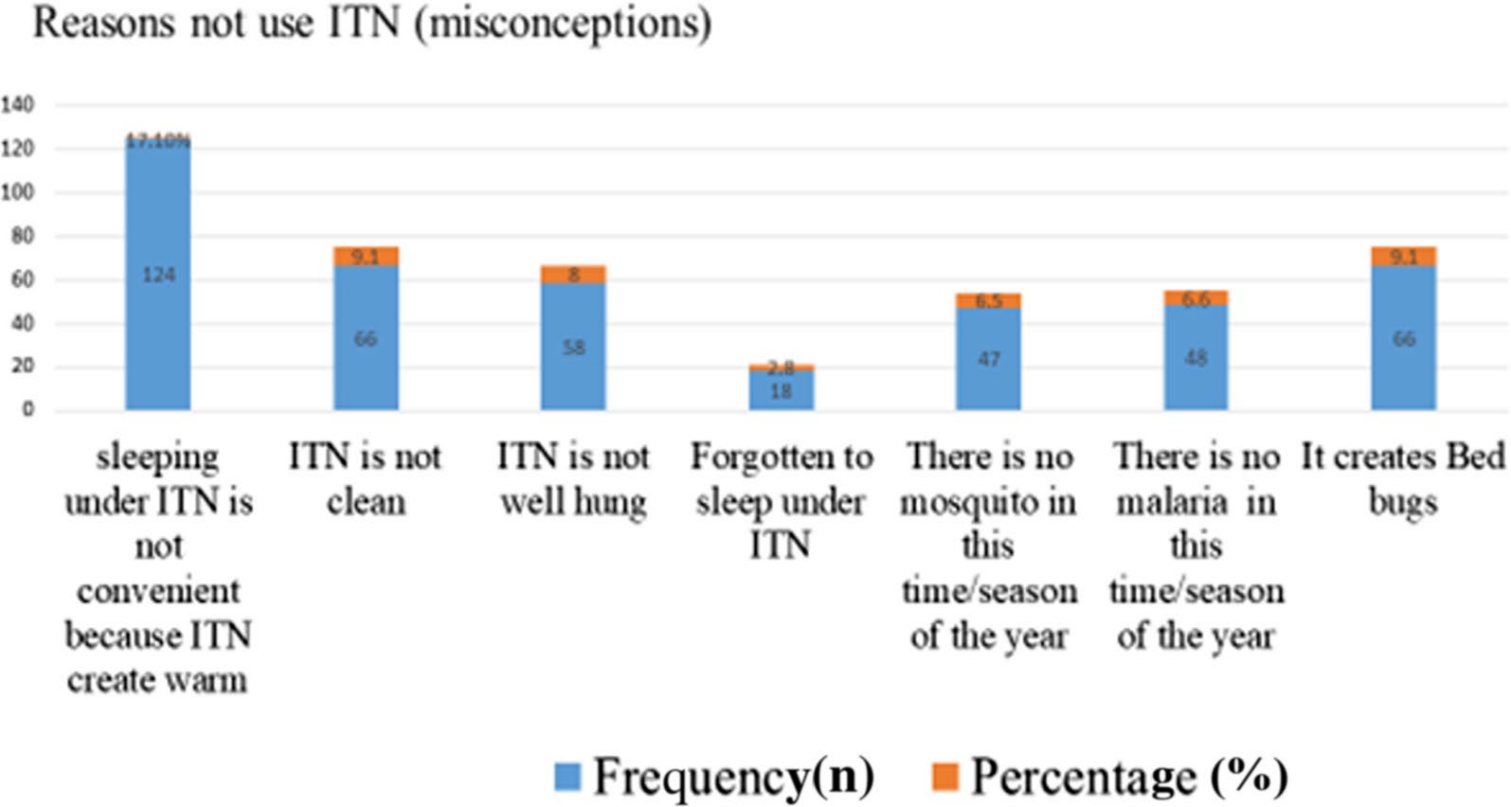
Reasons for not utilizing ITN among respondents in east Belessa district, northwest Ethiopia, 2020

About 286 (39.5%) of the participants reported that they did not sleep under ITN prior to the survey date. The top three reported reasons for not using ITN included: “sleeping under ITN is not convenient as it creates warm” (17.1%); “ITN create bed bugs” (9.1%); and “ITN are not clean” (9.1%) (Fig. 1).

#### Factors affecting ITN utilization

In the final model analysis, four variables including corrugated iron roof of the house (AOR = 1.53; 95% CI 1.15, 2.22), rural residence (AOR = 1.59; 95% CI 1.11, 2.28), ≥ 2 number of rooms in the house (AOR = 1.56; 95% CI 1.06, 2.30) and high level of perceived barrier (AOR = 0.53; 95% CI 0.38,0.74) have statistical significant assocaition with ITN utilization (Table 3).

**Table 3:** Factors associated with ITN utilization among pregnant women and under-five children, east Belessa District, northwest Ethiopia, 2020

Variables	ITN utilization		COR (95% CI)	P-value	AOR (95% CI)^a^	P-value
	Yes n (%)	No n (%)				
Roof of the house						0.03
Thatched-roof	73 (48.7)	77 (51.3)	1	0.03	1	
Corrugated iron	336 (58.5)	238 (41.5)	1.49 (1.04, 2.14)		1.53 (1.15, 2.22)	
Residence						0.01
Urban	85 (50)	85 (50)	1	0.05	1	
Rural	324 (58.5)	230 (41.5)	1.41 (1.00, 1.99)		1.59 (1.11, 2.28)	
Number of rooms in the house						0.02
1	305 (54.9)	251 (45.1)	1	0.11	1	
≥ 2	104 (61.9)	64 (38.1)	1.34 (0.94, 1.90)		1.56 (1.06, 2.30)	
Perceived barrier						0.00
Low	248 (62.3)	150 (37.7)	1	0.001	1	
High	165 (50.6)	161 (49.4)	0.60 (0.44, 0.79)		**0.53 (0.38, 0.74)**	

^a^14 variables, including age of respondents, roof of the house, residence, PW or UFC care givers’ educational level, number of rooms in the house, family size, marital status, level of knowledge, perceived severity, perceived benefit, perceived barrier, perceived susceptibility, self-efficacy and clues to action have been entered to the adjusted model

### Qualitative study results

#### Sociodemograhic characteristics of participants of the qualitative study

A total of 39 participants were involved in five FGDs. Each FGD had 7–9 participants. The participants ranged from 20 to 53 years of age. Sixteen (41%) of the FGDs participants had ever attended formal education. Majority (89.8%) of the FGDs participants were Orthodox Christians and the other 10.2% were Muslims. Thirty-two (82%) of the participants were housewives and the other seven (18%) were farmers.

A total of 5 participants were engaged in KII. All (100%) of the key informants were literate. The KII participants ranged from 26 to 32 years of age, two-fifth (40%) of them were Orthodox Christians, and all of them (100%) were governmental employees (Table 4).

**Table 4:** Demographic interviews

	Number of participants	Age years old	sex	
			M	F
FGD (urban WDA)	7	27–50		7
FGD (rural WDA)	9	20–38		9
FGD (urban PW and UFC)	7	21–38		7
FGD (rural PW and UFC)	9	20–38		9
Male FGD	7	30–53	7	0
5 FGDs (total participants)	39	20–53	7	32
KII	5	26–32		5
ALL	44	20–53	7 (15.9%)	39 (84.1)

#### Perceived severity and susceptibility of malaria

Malaria was widely perceived as a serious disease and recognized as a matter of community concern requiring community action in the district. Many participants believed that they were at risk of malaria infection and they believed that malaria is a serious disease and if it is left untreated that can lead to death.

“The most susceptible groups in the community are pregnant women and under-five children. This is because the pregnant mother provides nutrition for her baby due and then she may become weakened by this reason she may exposed easily to malaria. Malaria is a serious disease and a deadly disease if we do not take medication at the time” (25 years, grade 5, orthodox Christian women developmental army, Shamash kebele in FGD).

“We and children are highly vulnerable groups as compared to other segments of the population in the community. Especially we pregnant women are highly vulnerable because we give food and other minerals for our embryo” (38 years, grade 11, orthodox Christian pregnant women, Hamusit kebele in FGD).

“Malaria is a serious disease especially for children because they don’t take treatment. Even I treat my child up to go to Gamby hospital. Most vulnerable groups are children, pregnant women and mothers who have a neonate” (35 years, have no formal education, orthodox Christian under-five caregiver, Hamusit kebele in FGD).

In contrast, a few participants perceived that malaria is not a serious disease and they believed that it is treatable.

“I think we are vulnerable unless we use a bed net. We think that it is hard but some people may not think so” (Female KII, Gohalla).

“I think that they are vulnerable. Most of the time, when there are two or three children in our area, all of them do not use ITN properly. But they are aware that they are vulnerable and do not think that it can lead to death” (Female KII, Hammusit).

#### Perceived benefits of ITN utilization

Malaria prevention was the most frequently cited benefit of ITN utilization.

“Bed nets protects against mosquitoes, spiders and other debris” (27 years, have no formal education, Orthodox Christian, women developmental army, Gohalla kebele in FGD).

“We prevent malaria by using bed net and by keeping hygiene. Bed nets can protect from the bite of a mosquito. Not only this, but also it can protect from other foreign bodies” (30 years, no formal education, women developmental army, shamash kebele in FGD).

Protection against pests such as cockroaches, fleas, flies and spiders*,* and against snake bites was a very occasionally reported non-malaria related benefit of ITN utilization.

#### Perceived barriers of ITN utilization

Perceived barriers of ITN utilization were reported in three themes includng individual, socio-cultural, and institutional level barriers.

#### Individual level barriers

Most common individual-level barriers were related to misbeliefs and misconceptions towards ITN utilization. Participants claimed that they did not use ITN as they believed that it creates warm and bed bugs. Another individual level barrier of ITN utilization was participants’ preference of shapes of ITN. Some participants believed that God, not the ITN, prevents malaria.

*“*We use bed nets for various purposes in the sense that God saves us. Bed nets do not save us from malaria” (male FGD).

“Most of the time they said it creates bed bug and warm. And also they think that they may not acquire a disease if God does not permit” (female KII, Shamash kebele).

“It would be ideal if bed nets came in a circle rather than a rectangular shape. And it's good to have bed net every year” (female FGD, women developmental army).

“The most common barriers to ITN utilization in the community are: they believe that bed nets produce and gather bugs; they think that bed nets are hot; there is no malaria at this time and most people think that malaria occurs only in summer. Due to this, pregnant women and under-five children do not sleep under bed nets throughout the year” (female KII).

“We think that the bed net is poison for the newborns because their skin is very soft. Due to this, most of the time people say do not sleep newborns under bed net” (women developmental army FGD).

The majority of FGD and KII participants mentioned that ITN was used for other purposes (i.e., for donkey loads, straw strips, camshaft, straw assembly and straw storages, and vegetable spinning rather than malaria prevention).

“ITN is used for straw assembly and straw for storage. If it is over, we will go ahead and use it for carrying water” (Female FGD, WDA).

“They use it for many purposes for other purposes than expected e.g. straw, rope, and padding” female KII, Woreda malaria officer).

#### Institutional level barriers

Most common institutional level barriers raised by the participants were: insufficient ITN accessibility, lack of timely immersion of ITN in the insecticide chemicals, failure to allocate ITN proportional to household’s family size, and failure to give vulnerable groups priority.

“No use of ITN because bed net is not accessible, so I have seven family members, for example, I received only two” (Male FGD).

“I also use bed net with my kids but there are people who do not use it as a family because bed nets are inadequate” (female FGD, WDA).

“All family members do not sleep on ITN because the bed net is not accessible for in family size” (Female FGD, WDA).

“Lack of accessibility to family size and there is no awareness on rinsing of bed net at a time” (Female FGD, Pregnant women, and under-five child-caregiver).

“The barriers that raised from the community is that lack of chemicals in the bed net” (male FGD).

#### Socio-cultural level barriers

Most common socio-cultural level barriers cited by the participants include custom of ITN utilization for other purposes rather than malaria prevention, lack of adequate sleeping places, and cultural misbelieves towards ITN (i.e., participants believed that ITN 'create' bed bugs).

“The bed net is used to protect against spiders, flies and mosquitoes. Those who do not have a shelter will sleep on the ground floor and will not use it” (female FGD, women developmental army).

“All children in my family do not sleep under bed net because the bed is one. Due to this, some of the children sleep on the floor. For this reason, some family members sleep without ITN because it is not comfortable to stretch on the floor” (27 years, grade 9, orthodox Christian women developmental army, Hamusit kebele).

“Now there are a lot of gaps in bed net usage throughout the year. We need to use bed net throughout a year but they do not use it because they think it causes warmth and produces bed bugs” (Female KII, Woreda Malaria officer).

“Because of the size of the number of people live in the household, there is no bed for the whole family. Most of the family’s sleeping space is one with more than four families. Due to this, most families sleep on the floor” (female KII, achikan kebele).

### Self-efficacy and cues to action

Most participants in the qualitative study stated that they were confident in their ability to use the available ITN. Receipt of a free ITN was commonly seen as a benefit.

“We had people who discontinued using bed nets. As we reported the issue to the office, they were offered some bed nets. We have addressed at the household level. The plan was to distribute about 1150 bed nets. But 1650 bed nets were distributed. So, the coverage is more than 100%” (female KII Hamusit health extension).

All key informants stated that high community’s coverage with ITN would contribute to a reduction in malaria transmission. All participants reported that the sources of malaria information (cues) are health professionals at the health centre and health post and the health development army at the community.

## Discussion

This study was conducted to assess the prevalence, associated factors and barriers of ITN utilization PW and UFC in east Belessa district. The study poses its own strength and limitation. The study employed a mixed community-based design and behavioural models. In addition, direct observation was done to verify participants’ self-reported information regarding ITN utilization. As the study was conducted during the dry season when the malaria transmission is low, ITN utilization might be underestimated.

The prevalence of ITN utilization was low and independently predicted by an iron corrugated roof of the house, rural residence, ≥ 2 numbers of rooms in the house and high level of a perceived barrier. Its main barrier was related to misconceptions towards ITN. In the current study, about 56.5% of the participants utilized ITN. This figure is comparable with the findings of the studies done in Uganda, 56% [28], and Myanmar 52% [29]. However, the magnitude of ITN utilization is higher than what was reported from Adama, Ethiopia, 34.9% [30]. It is also higher then compared to findings of the studies done in Nigeria, 45.3% [31], and two studies in Ghana, 34.4 and 41.7% [32, 33]. The possible reason for such discrepancy could be related to variation in ITN ownership coverage. The proportion of participants owing ITN in the current study is higher than what was reported in the previous studies. For example, about 100% of respondents in the current study owned at least one ITN. However, only 59.7%, 52% and 70.3% of the respondents owned ITN in Adama, Nigeria and Ghana, respectively. In this perspective, existing evidence suggest that higher ITN ownerships coverage is directly proportional to better ITN utilization in a given area [30–33]. Moreover, the higher ITN utilization in the current study could be partly explained through participants’ good knowledge of malaria and ITN. For instance, 72.1% of the respondents had a good level of knowledge on ITN and malaria. Similarly, 97.5% of the respondents in the current study reported that ITN utilization could prevent malaria. In fact, people with better knowledge on malaria and ITN are likely to adhere to malaria prevention guidelines including ITN utilization. Therefore, stakeholders need to strengthen creating community awareness towards malaria and ITN.

However, the figure of the current study is lower than the results reported from other studies conducted in Ethiopia, such as the national coverage of ITN utilization among pregnant women (70%) and under-five children (74%) [27], Benishangul Gumuz Community (80.7%) [34], and Addis Zemen Hospital (74.3%) [35]. It is also lower compared with the reports from Democratic Republic of the Congo (78.4%) [23]. The inconsistencies could be attributed to variation in measurement of the outcome variable: the current study employed respondents’ self-report of ITN utilization followed by direct observation of hanged ITN. Therefore, direct observation could avoid false self-reports, thereby it could prevent overestimate of ITN utilization compared with studies reported entirely by self report. For example, the study done in Addis Zemen measured the outcome variable by interview alone which might be subjected to social desirability bias. Consequently, the reported magnitude of ITN utilization could be overestimated [35]. Another possible reason for the lower observed ITNs utilization in the current study could be connected to the season when the investigation was undertaken. This study was carried out during the dry season, when participants tend to be negligent in using ITN as they would perceive no risk of malaria in the dry season. This idea was corroborated by the findings of the qualitative study. Furthermore, the lower ITN utilization could be related to participants’ misperceptions towards malaria and ITN as evidenced by the vast majority results of HBM and qualitative study. These results imply that the level of misperception of participants towards malaria and ITN was sufficient to preclude ITN utilization, and call for more efforts to be applied to reverse the community’s misconceptions towards malaria and ITN, through IEC and BCC. In addition, sufficient and sustainable free ITN distribution by considering family size of the households would be helpful in escalating ITN utilization as the qualitative results demonstrate the existence of disproportional and insufficient accessibility of ITN despite a 100% distribution of ITN to household ratio.

The multivariable logistic regression analysis result showed that the odds of ITN utilization were higher among respondents; living in the house with iron corrugated roof, residing in the rural, having a house with ≥ 2 number of rooms and having low perceived barriers. However, there is a need for caution against drawing causality as this study was cross-sectional in nature. The respondents having corrugated iron house roof were 1.53 times more likely to utilize ITN compared with the reference group. This association is plausible since houses with corrugated iron roofs are relatively more suitable to hang nets and may have enough sleeping areas. This idea is also supported by the qualitative findings. Most FGD participants reported that lack of appropriate sleeping place was one of the major barriers of ITN utilization. The findings of the current study suggest the need of providing support, encouragement and information to the residents of the district so as to build their houses in a convenient manner for ITN utilization. However, since as a substantial proportion (80%) of the participants had corrugated iron houses, this figure must be considered while interpreting the association between this variable and the outcome.

In the current analyses, rural residence was significantly and positively associated with ITN utilization. Accordingly, the odds of ITN utilization were 1.59 times higher among the rural residents than urban dwellers. The finding is in accordance with the study done in Arbaminch, southeast Ethiopia [36]. This could be due to the fact that most rural villages in Ethiopia are established around water sources such as rivers. In addition, Ethiopian Government has built dams in the rain deficient rural valleys. These stagnant water sources could create a favourable condition for mosquito breeding as said by a male FGD participant. The current study exhibited that ITN utilization was independently predicted by the number of rooms in a house. Participants living in the house with at least two rooms were 1.56 times more likely to use an ITN as compared to those respondents who lived in the one-roomed house. The finding is corroborated by the results of previous studies done in Mirab Abaya District [22].

Participants with a high level of the perceived barrier were 47% less likely to use an ITN as compared to those respondents with a low level of a perceived barrier. A statistically significant association between high level of perceived barrier and low ITN utilization implies that stakeholders need to probe, listen and work on participants’ perceived barriers of ITN utilization as such barriers are significant enough to compromise ITN utilization among study participants.

This study also explored the barriers of ITN utilization. Based on the findings of the qualitative study, almost all barriers of ITN utilization in the district were related to misconceptions and misperceptions towards malaria and ITN. In connection to this, the analysis of HBM shows that nearly half (45%) of the subjects have low perceived barrier [16]. These barriers are similar to what was reported in the studies done in Ghana, Uganda and Kenya [28, 37, 38]. These findings clue an important implication that stakeholders should work by far collaboratively and exert more efforts to design and apply appropriate interventions, such as Information, Education and Communication (IEC), and Behavioural Change Communication (BCC) towards malaria and ITN.

## Conclusion

The prevalence of ITN utilization in the study area was lower than the national target and its main barriers were related to misconceptions and misperceptions towards ITN and malaria. Corrugated iron house roof, ≥ 2 number of rooms and rural residence were factors positively associated with ITN utilization. In addition, high perceived barrier was negatively associated with ITN utilization. Therefore, reversing the community’s misconceptions towards malaria and ITN through IEC and BCC would improve ITN utilization.

## Data Availability

The datasets employed in the current study can be available from the corresponding author upon the reasonable request.

## Abbreviations

AOR: Adjusted odds ratio
BCC: Behavioral change communication
COR: Crude odds ratio
EDHS: Ethiopian demographic health survey
FGD: Focus group discussion
HBM: Health belief model
IEC: Information, education, and communication
ITN: Insecticide-treated net
KII: Key informant interview
PW: Pregnant women
RHB: Regional, health, bureau
UFC: Under five children
WHO: World Health Organization
